# *Cleome gynandra*: A wonder climate-smart plant for nutritional security for millions in semi-arid areas

**DOI:** 10.3389/fpls.2022.1003080

**Published:** 2022-09-23

**Authors:** Chuene Victor Mashamaite, Alen Manyevere, Ereck Chakauya

**Affiliations:** ^1^Department of Agronomy, University of Fort Hare, Alice, South Africa; ^2^AUDA-NEPAD Centre of Excellence on Science Technology and Innovation, (AUDA-NEPAD CoE STI), Stellenbosch University, Stellenbosch, South Africa; ^3^Southern Africa Network for Biosciences (SANBio), Council for Scientific and Industrial Research, Pretoria, South Africa

**Keywords:** *Cleome gynandra*, climate change, underutilised crops, modern biotechnology, gene sequencing, malnutrition, COVID-19, crop productivity

## Abstract

Spider plant (*Cleome gynandra*) is predominantly used as a traditional leafy vegetable throughout Africa and is considered a rich natural source of essential nutrients such as vitamins, minerals and proteins. With the increase in malnutrition, diet related non-communicable diseases and poverty across the continent of Africa, the spider plant is a *bona fide* alternative healthy food crop to alleviate these challenges. Spider plant is an erect annual herb that could grow up to 150 cm tall, strongly branched, with a long taproot and few secondary roots. It is commonly consumed in resource-poor communities especially during times of major food scarcity. It is a drought-tolerant and resilient annual vegetable crop capable of growing well in a wide range of climatic and edaphic conditions. Despite the potential benefits and wide adaptability, progressive attempts towards the development of *C. gynandra* as a crop have been impeded by issues like low investment in research and development resulting in poor seed quality, relatively low yields and susceptibility to pests and diseases. In this paper, we reviewed the research that has been done regarding its morphology, growing conditions, production and utilisation (i.e., nutrition). The current review highlighted the status of the science in advancing the domestication of *C. gynandra* as a potential power crop for several African countries. The review concluded that with the advancement of modern biotechnology techniques and genome sequencing, there is a compelling case for investment and development in *C. gynandra* as a candidate for managing micronutrient deficiencies during the post-pandemic era. Finally, the existing knowledge gaps (e.g., breeding) that necessitate explorations were identified and recommendations that could enhance its development and potential commercialisation were made.

## Introduction

Increasing demand for food and rising cases of malnutrition are growing global challenges which are expected to worsen in developing nations due to the corona virus (COVID-19) pandemic and current global conflicts (Chang et al., 2018; FAO et al., 2021). According to the United Nations (2019), the world population reached 7.7 billion in mid-2019 and is anticipated to reach 8.5 billion by 2030. As such, ensuring food supply and security for the growing population becomes one of the key global challenges. Currently, the world is facing the double burden of undernutrition (thinness, stunting and underweight) and overnutrition (obesity and overweight) (Thow et al., 2018). The 2021 Global Nutrition Report estimated that 149.2, 45.4 and 38.9 million children under five years are stunted, wasted and overweight, respectively. About 2.2 billion (40%) of both men and women are reported to be obese or overweight (Development Initiatives, 2021). In sub-Saharan Africa, the number of malnourished people increased by 12% in the last five years to 250 million people and it is predicted that the number of undernourished people will increase by a further 7.4% in this region (FAO et al., 2020). Moreover, the situation may be worsened by the effects of the COVID-19 pandemic.

Although, it is too early to quantify, the 2021 Global Nutrition Report estimates that about 155 million people worldwide have been pushed to extreme poverty allegedly due to the pandemic (Development Initiatives, 2021). Furthermore, excessive costs of nutritious food together with constant rising levels of income inequality have placed healthy diets out of reach for over three billion people worldwide (FAO et al., 2021). The most vulnerable groups affected by this are mainly women and children as well as impoverished people living in marginal communities. In contrast, the consumption of poor-quality diets results in micronutrient deficiencies, and consequently increases diet-related non-communicable diseases and obesity (Willett et al., 2019). Concerns regarding environmental degradation, loss of biodiversity and climate change have prompted a call to rethink the current configuration of the food system (Drimie and Pereira, 2016). Indeed, changing climate has put more burden on the already constrained natural resources by reducing the resilience of agro-ecosystems that produce food (Mabhaudhi et al., 2019a). Thus, causing the rapid rise in hunger, food insecurity, malnutrition, and diet-related medical conditions (Wheeler and von Braun, 2013).

Within the Southern African Development Community (SADC) region, 23% and 3% of children under the age of five years were stunted and wasted in 2020, respectively (FAO et al., 2021). These were ascribed to malnutrition because their food lacked essential micronutrients (i.e., zinc, iron, vitamins, iodine and folate) (SADC, 2020a). South Africa has also been affected by nutrient deficiencies that cause many health conditions, particularly in children (27%) (Shisana et al., 2013; UNICEF 2020). This is mostly attributed to household food and nutrition insecurity, poverty, lack of adequate health care facilities and infrastructure (UNICEF, 2020). Other report has shown that the problem of obesity and overweight still exist in the SADC region, especially in South Africa, Botswana and Lesotho (SADC, 2020b). According to the 2016 South African Demographic and Health Survey (SADHS), one out of five adults in South Africa was obese (especially in women), and 13% of children below five years were overweight (Department of Health et al., 2017). Schönfeldt et al. (2010) and Govender et al. (2021) deduced that high obesity levels recorded in South Africa could be ascribed to lack of physical activities and excessive consumption of foods with high content of sugar, salt and fats.

Among 30 thousand edible plants, only 103 crops account for 90% of the global foods (FAO, 2019) and wheat, rice and maize are the three main crops accounting for over 50% of the plant-based human diet (FAO, 2018). Therefore, there is a need for urgent extensive use of locally available, highly adaptable underutilised crops to diversify healthy food sources (Mustafa et al., 2021), especially with the current global pandemic and climate change crisis. The World Health Organisation (WHO) global call has emphasised that for any country to ensure effective and sustainable food production, research and developments must be focused on local foods and feeding practices (WHO, 2020). According to Mustafa et al. (2021), it is critical to incorporate strategies that emphasise on alleviating poverty, malnutrition and non-communicable diseases, particularly now as the current COVID-19 pandemic persist. As a result, various strategies such as cultivation, biofortification and the promotion of underutilised indigenous crops are alternative suggestions to diversify diets among economically marginalised communities (Govender et al., 2021). Focus on reinvigorating underutilised indigenous and traditional crops and creating their market, has been perceived as an entry point for improving diets and improving their sustainability (Mabhaudhi et al., 2017; Aworh, 2018; Govender et al., 2021).

Traditional leafy vegetables are ‘wild vegetables’ usually collected by poor households from the bush or fallow fields. They are known to rural dwellers in some African communities as ‘*morogo’* or ‘*imfino’* (Aworh, 2015) and these indigenous leafy vegetables have formed part of day-to-day livelihood among Africans for centuries (Van Rensburg et al., 2007a; Mabhaudhi et al., 2017). However, the displacement of indigenous crops by a few major crops has inevitably contributed, in part, to the limited success of the global food systems (Mbhenyane, 2016; Coq-Huelva et al., 2017; Mabhaudhi et al., 2019b). With a growing shift towards healthy diets, underutilised functional food crops are now gaining popularity due to their nutritional, pharmacological, therapeutic and nutraceutical benefits (Mudau et al., 2022). There are hundreds of traditional wild leafy vegetables that are of agricultural and nutritional significance in Africa and have the potential to add diversity to traditional diets by providing unique flavours, textures and other sensory attributes (Uusiku et al., 2010). Some indigenous vegetables that have caught research attention in recent times include *Amaranthus hybridus* (amaranth), *Bidens pilosa* (blackjack), *Citrullus lanatus* (bitter melon), *Cleome gynandra* (spider plant), *Solanum scabrum* (nightshade) and *Vigna unguiculata* (cowpea) (Mazike et al., 2022). Among these underutilised leafy vegetables, the scope of this review is limited to a functional food crop, *C. gynandra* (synonym: *Gynandropsis gynandra*). This is because, despite its exceptional multifunctional benefits, including nutritional and medicinal attributes that are important in maintaining good health and protection against diseases; it is still underutilised.

*Cleome gynandra* with both nutritional and medicinal properties may contribute to the attainment of nutrition, food security and wellbeing (Moyo and Aremu, 2022). Its drought tolerance combined with high nutritional value makes this vegetable ideal for cropping systems in water-scarce regions (Cernansky, 2015) where drought occurrences are expected to rise because of changing climate (Frischen et al., 2020). Therefore, it is important to promote the production and utilisation of this drought tolerant crop with high nutritional value as a means of mitigating climate change as well as ensuring nutritional security (Mazike et al., 2022). In this paper, we emphasise the need to develop management practices of this leafy vegetable and encourage its cultivation and utilisation. We reviewed the research that has been done regarding its morphology, growing conditions, production and utilisation (i.e., nutrition). Furthermore, we identified existing knowledge gaps (e.g., breeding) that need to be addressed and made recommendations that could enhance its use, development and potential commercialisation.

## *Cleome gynandra* highly neglected but found across tropical and subtropical regions

*Cleome gynandra* belongs to the Cleomaceae family and is indigenous to sub-Saharan Africa (SSA) and South-east Asia (Bala et al., 2010; Munene et al., 2018; Shilla et al., 2019). In SSA, it is particularly found in many countries which includes Botswana, Kenya, Malawi, Namibia, South Africa, Tanzania, Uganda, Zambia and Zimbabwe (Vorster et al., 2002; Mauyo et al., 2008; Onyango et al., 2013; Jinazali et al., 2017; Omondi et al., 2017; Chataika et al., 2020). Additionally, it has been found growing naturally in seven of the nine South African provinces, which are Limpopo, Northwest, Gauteng, Mpumalanga, KwaZulu-Natal, Free State and Northern Cape (Mishra et al., 2011; Mabhaudhi et al., 2017). It is commonly known as *‘lerotho*’ in Sepedi; ‘*murudi*’ in Tshivenda; ‘*amazonde*’ in isiZulu; ‘spider flower or plant’, ‘cat’s whiskers’ and ‘African cabbage’ in English (Van Wyk and Gericke, 2000; Vorster et al., 2002). National studies conducted in South Africa has indicated the worsening trends of vitamin A deficiency since 2005 (Shisana et al., 2013). *Cleome gynandra*, being a rich source of vitamins and minerals (Vorster et al., 2002), can contribute to alleviation of malnutrition by providing a healthy and affordable nutrient-dense diet. However, there is a decline in consumption of *C. gynandra* in Africa due to the poor availability, dearth of knowledge on the nutritional content, and negative perception as it is collected from the wild and is partially cultivated (Maseko et al., 2017).

## Morphological description of *C. gynandra*

**Figure 1:**
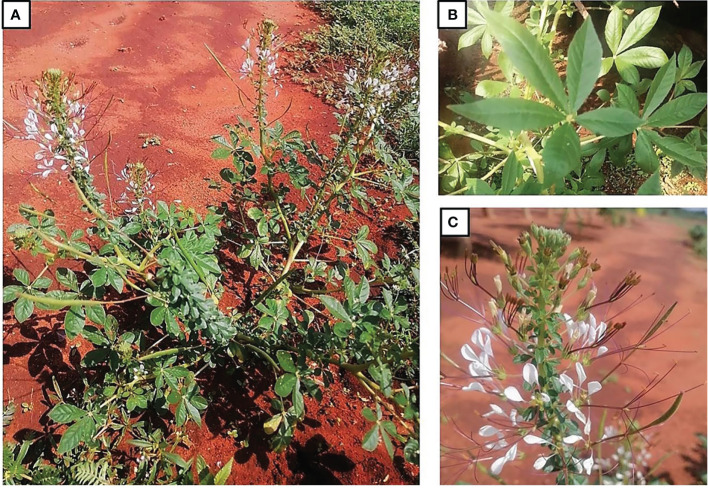
Traditional leafy vegetable, *Cleome gynandra*, growing at Genoa village in the Limpopo Province, South Africa. **(A)** whole plant; **(B)** leaves; **(C)** flowers (Photos by Mashamaite CV).

*Cleome gynandra* is an annual, herbaceous, erect and branched plant that can grow up to 1.5 m tall (Vorster et al., 2002). The leaves are compound and palmate with three to seven leaflets (**Figure 1**). The stem is granular and hardly hairless, with longitudinal parallel lines (**Figure 1**). It contains numerous branches which becomes woody as the plant ages (DAFF, 2013; Shilla et al., 2019). The pigment on the stems varies from green to pink and purple (Van Rensburg et al., 2007b; Mishra et al., 2011). The terminal inflorescences have distinct small white, lilac or pink coloured flowers (Schippers, 2002). The flowers are attached to the stem by short, equal stalks at equivalent distances (DAFF, 2013). The fruits of *C. gynandra* are tiny and siliques, while the seeds are brown and circular with rough seed coat and parallel longitudinal lines (Van Wyk and Gericke, 2000; DAFF, 2013). The plant has a long taproot and a small number of secondary roots covered with root hairs (DAFF, 2013; Shilla et al., 2019).

## Growing conditions and cultivation of *C. gynandra*

Although there is limited literature on production and cultivation practices regarding several African traditional leafy vegetables like *C. gynandra*, there is a common claim that these crops require minimum water and grow well in marginal soils (Aworh, 2015). Furthermore, there are assertions that these leafy vegetables can achieve better yield without application of production inputs such as fertilisers, irrigation, pesticides and herbicides (Aworh, 2014; Aworh, 2015). This leafy vegetable is propagated from seeds (DAFF, 2013) and it can thrive in fragile and marginal conditions including dry lands, degraded lands and wetlands (Baldermann et al., 2016). For example, the plant grows in the wild, crop fields, on mountain slopes and household yards in the rural Limpopo Province (Faber et al., 2010). In addition, they are more resistant to pests and diseases, drought and other unfavourable growing conditions when compared to commercial vegetables (Mabhaudhi et al., 2019a). Hence, lands that are no longer fit for cultivating commercial crops could be suitable for the cultivation of traditional leafy vegetables like *C. gynandra* (Mabhaudhi et al., 2017).

### Prospects of *C. gynandra* as a climate smart crop

Continuous rising in global temperatures as a result of climate change prompts severe weather events such as drought, flooding and heat waves (Feulner, 2017). These challenges are causing socio-economic and health instabilities (Schmidhuber and Tubiello, 2007). Climate shocks will impact the affordability and availability of nutritious healthy diets; making them out of reach for vulnerable and marginalised communities (Campbell et al., 2016). It has been estimated that nutrition and food security of about 70% of people in Africa, Asia and Asia-Pacific will be negatively affected due to persistent climate change (SADC, 2020a). Therefore, an adaptation of climate-smart agriculture will safeguard a sustainable increase in crop productivity to meet the pervasive food demand (Munaweera et al., 2022). The use of locally available, underutilised crops such as *C. gynandra* can provide diverse sustainable and healthy diets, while at the same time contributing to climate change mitigation strategies (Bvenura and Afolayan, 2015).

*Cleome gynandra* plants adapt well to diverse habitats (predominantly in warm climates) due to their C4 photosynthetic mechanism (Raju and Rani, 2016). Mishra et al. (2011) revealed that this photosynthetic mechanism and pathway allows plants to thrive in hot and drier environmental conditions including semiarid, humid and subhumid climates with diverse soil types. The C4 photosynthetic pathway uses primary CO_2_-fixing enzyme, phosphoenolypyruva carboxylase for distilling CO_2_ close to ribulose-1,5-biphosphate carboxylase/oxygenase; thus, enabling effective carbon assimilation (Sommer et al., 2012). It is reported that C4 plants are more resistant to the impacts caused by high CO_2_ on nutrient density than C3 plants like wheat and rice (Medek et al., 2017). These species exhibit high-level of photosynthetic efficiency under favourable climatic (i.e., temperature and light intensity) and soil moisture conditions (Kwarteng et al., 2018). Due to ever-increasing atmospheric CO_2_ levels and elevated temperatures, promotion, distribution and use of C4 species could be on the rise soon (Sage, 2004). According to Moyo and Aremu (2022), investigations aiming at explicating the physiological and molecular mechanisms of C4 photosynthesis on *C. gynandra* could provide more information on regulation of photosynthesis. It is important to elucidate since this process directly affect the productivity of crops.

*Cleome gynandra* can grow in regions with short periods of valuable rainfall and is highly susceptible to flooding (DAFF, 2013). Though the plant can tolerate some degrees of drought, severe water stress may reduce leaf yield and quality (Van Rensburg et al., 2007b). Due to its tropical origin, *C. gynandra* requires temperatures that range between 18°C and 25°C (Mishra et al., 2011; DAFF, 2013). This leafy vegetable grows well above the sea level of up to 2400 m since it prefers complete exposure to sunlight and performs poorly when shaded (Modi and Mabhaudhi, 2013; Chand et al., 2022). The deep root system is established first during plant growth of *C. gynandra*, followed by foliage development. The leaves display the circadian movement where they follow the direction of the sun (Kwarteng et al., 2018).

Low temperature is one of the imperative plant abiotic stress factors which restrict plant growth, development and productivity. *Cleome gynandra* does not grow and develop well under temperatures below 15°C (Modi and Mabhaudhi, 2013). Chilling temperatures cause several metabolic and physiological disturbances in cells of plants that are susceptible to low temperatures. Chilling stress results in injury and even death of tropical and subtropical plant species like *C gynandra* (Jouyban et al., 2013; Batool et al., 2020). According to Van Rensburg et al. (2007b), the plant grows best during summer and is sensitive to freezing conditions. Since *C. gynandra* is widely distributed worldwide, it can therefore be a suitable plant species for restoring ecologically degraded and warm environments (Raju and Rani, 2016; Shilla et al., 2019).

### Cultivation of *C. gynandra*

*Cleome gynandra* is adapted to various soil types, ranging from sandy to clay loams and requires soil pH that range between 5.5 and 7.0 (DAFF, 2013). This leafy vegetable favours well-drained, medium-textured soils and is highly susceptible to poorly drained or heavy clay soils (Shilla et al., 2019). The study of Schippers (2002) indicated that the application of fertilisers containing a substantial amount of nitrogen increased the leaf number and size. Mauyo et al. (2008) indicated that applying nitrogen fertiliser at the rate of 80 kg.ha^-1^ increased plant height, number of leaves and fresh leaf of *C. gynandra*. Others demonstrated that farmyard manure was used to enhance the growth and yield of this leafy vegetable (Ng’etich et al., 2012). According to Kumar et al. (1984), this plant has been empirically proven to tolerate salt and water stress, which is important for soil erosion control. Even though *C. gynandra* produce well with adequate water supply due to its ability to tolerate certain levels of water stress; nevertheless, prolonged moisture stress could delay flowering and senescence stages (Van Rensburg et al., 2007b). Despite these advantages, indigenous vegetables like *C. gynandra* often produce low economic yield and are less productive when compared to commercial vegetable cultivars (Moyo and Aremu, 2022).

The seeds of *C. gynandra* enter physiological dormancy for 4 – 5 months after harvest and only germinate after 6 months of harvest (Chweya, 1990; Ramphele et al., 2020). The process of germination important in the domestication of crops and this is because lack of uniform seed germination can lead to poor seedling establishment and subsequently affect total yield (Kwarteng et al., 2018). Several studies attempted to break dormancy of *C. gynandra* seeds using different methods. For example, Kamotho (2004) demonstrated that storing freshly harvested seeds for duration of one year could break dormancy; however, it was specified that seed storage conditions determine the success of breaking the dormancy. The effect of temperature, light and pre-germination treatments were tested on *C. gynandra* seeds, and it was revealed that alternating temperatures of 20 – 30°C under dark conditions produced high germination percentages (Ochuodho, 2005; Ochuodho and Modi, 2005; Ochuodho and Modi, 2007). Ochuodho and Modi (2005) and Tibugari et al. (2012) made recommendations of puncturing the dormant seeds at the radicle to improve germination as the most effective pre-treatment method. The seeds of *C. gynandra* are negatively photoblastic and exposure to light for longer periods of 12 hours per day will reduce their germination due to photo inhibition (Ekpong, 2009; Raboteaux and Anderson, 2010). Others indicated that treating seeds with plant growth regulators like gibberellic acid was also found to be effective in breaking dormancy by disrupting metabolic processes that impede seed germination (Bewley and Black, 1994).

*Cleome gynandra* plants have a short growth period that enables them to avoid late emerging stresses (Shilla et al., 2019). In many countries across the world, it is not formally cultivated, but people living in rural areas occasionally plant the natural weedy population; either by broadcasting seeds or practising selective weeding (Hart and Vorster, 2006). Most communities harvest this leafy vegetable from their homestead for consumption purposes (Thovhogi et al., 2021). It can be harvested in diverse ways when it reaches a height of about 15 cm by uprooting the entire plant, topping, cutting back to ground level or picking individual leaves or leafy branches at frequent intervals (Hart and Vorster, 2006; Faber et al., 2010). Regular picking and deflowering improve lateral growth and this prolongs the harvesting period. Harvesting of leaves usually commences from four to six weeks after seedling emergence and can last up to five weeks (Chweya, 1990; Shilla et al., 2019). The seeds are harvested when pods are completely ripe and yellow but prior to opening naturally to avoid shattering (DAFF, 2013). However, increased harvesting of *C. gynandra* from the wild without proper cultivation could be unsustainable (Maseko et al., 2017). Therefore, it is necessary to conduct further investigations on the cultivation and harvesting of this crop to create basic production guidelines.

## *Cleome gynandra* as a nutritional food source

*Cleome gynandra* is one of the leafy vegetables that have high potential for development as a future crop, particularly in the tropical and subtropical regions where it grows naturally (Vorster et al., 2002; Mabhaudhi et al., 2017). The plant has many uses including food and medicinal purposes for humans, animal feed and plant protectant potential. However, the scope of this review is only limited to its potential as a nutritional food source. *Cleome gynandra* is an important leafy vegetable for attaining household food security in rural areas of many African countries (Van Wyk and Gericke, 2000; Vorster et al., 2002; Mabhaudhi et al., 2017). The leaves possess dietary polyphenolic phytochemicals that promote health and include flavonoids, essential ions, polyphenols and terpenoids (Chataika et al., 2022; Moyo and Aremu, 2022). Other compounds that can be utilised as food supplements or as flavouring and colouring agents as well as for extending shelf-life of various products are also present in *C. gynandra* leaves. These include ascorbic acid, violaxanthin, α and β-carotene, α, β, and γ -tocopherol, β-cryptoxanthin and luteolin (Chand et al., 2022). The oil content of the seeds contains polyunsaturated fatty acids which have high health benefits, and this oil can also be extracted by hand pressing (Mnzava, 1990).

It is the cheapest and most accessible rich source of vitamins (A and C), protein (23.4%), fibre (8.3%), and essential minerals (Uusiku et al., 2010; Aworh, 2015). The study by Moyo et al. (2018) showed that *C. gynandra* leaves possess higher concentrations of calcium, iron, phosphorus, potassium and vitamin C when compared to commercial vegetables like *Brassica oleracea* var. capitata (cabbage) and *Beta vulgaris* L. (Swiss chard). In **Table 1**, we showed that a significant contribution with regards to the recommended dietary allowance (RDA) could be made by consumption of lower doses of *C. gynandra* leaves for healthy adults. The RDA is the total intake of energy and dietary components which are considered adequate for the maintenance of wellbeing of an individual (FAO/WHO, 2002). Itanna (2002) expressed that people’s health can decline if they do not follow the RDA; but bearing in mind that excess uptake may also lead to other health problems.

**Table 1:** Contributions of *Cleome gynandra* leaves to recommended dietary allowance of the selected nutrients for healthy adults aged between 19 and 59 years.

Nutrients	RDA (mg/day)	Source	*C. gynandra* leaves (mg.100g^-1^)	Source	% Contri.
Vitamin A (β-carotene)	0.8^a^; 1.0^b^	NRC, 1989	6.7–18.9	Mishra et al., 2011; Jinazali et al., 2017	837–2363^a^; 670–1890^b^
Vitamin B_2_	1.1^a^; 1.3^b^	FAO/WHO, 1998	0.08–0.1	Van der Walt et al., 2009; Schönfeldt and Pretorius, 2011	7–9^a^; 6–8^b^
Vitamin C (Ascorbic acid)	60	NRC, 1989	127–484	Mishra et al., 2011; Jinazali et al., 2017	212–807
Protein	750	FAO/WHO, 1998	2.6–6.0	Odhav et al., 2007; Mibei et al., 2011; Schönfeldt and Pretorius, 2011; Jinazali et al., 2017	0.4
Calcium	1000	FAO/WHO, 2004; Koubova et al., 2018	2209.8	Jinazali et al., 2017	221
Iron	15	NRC, 1989	35.7	Jinazali et al., 2017	238
Zinc	12^a^; 15^b^	NRC, 1989; FAO/WHO, 2004	8.4	Van der Walt et al., 2009	70^a^; 56^b^
Potassium	2000	NRC, 1989; Sodamade et al., 2013	410	Van den Heever and Venter, 2007	21
Magnesium	280^a^; 350^b^	NRC, 1989	86	Van den Heever and Venter, 2007	31^a^; 25^b^
Sodium	1900^a^; 2100^b^	NRC, 1989; Sodamade et al., 2013	34	Van den Heever and Venter, 2007	1.8^a^; 1.6^b^
Phosphorus	800	NRC, 1989; FAO/WHO, 2002	12	Chweya, 1990	1.5
Manganese	5	NRC, 1989	10–37.5	Odhav et al., 2007; Glew et al., 2009; Maroyi, 2013	200–750
Copper	1.5	NRC, 1989	2–8	Odhav et al., 2007; Glew et al., 2009; Maroyi, 2013	133–533

% Contri. = (Nutrient content of leaves ÷ RDA) ×100; ^a^ denotes females; ^b^ denotes males. Table legend: Percentage contributions are shown by (% Contri.) and recommended dietary allowance is denoted with (RDA).

Beal et al. (2017) estimated that about one-third of the world’s population was affected by iron and zinc deficiencies. About 30% of the world population was anaemic, and 50% of such cases are caused by iron deficiency (IGN, 2019). Little efforts have been made to reduce cases of anaemia since 2012 and this calls for urgent solutions globally since 29.9% of women aged 15 to 49 years were affected by anaemia in 2019 (FAO et al., 2021). According to WHO, 40% and 42% of pregnant women and children below the age of five years, respectively, are currently anaemic (WHO, 2021). This global challenge of Fe and Zn deficiency can be reduced by incorporating nutrient-dense leafy vegetables such as *C. gynandra* into our diets (Stangoulis and Knez, 2022). In addition to antibiotics, COVID-19 patients are given zinc and vitamins to boost their immune system and shorten the number of days the common flu lasts. Given that *C. gynandra* is rich in vitamins A and C, as well as zinc and iron, it could serve as one of the alternatives to prevent anaemia while reducing the severity of COVID-19 symptoms. This provides a compelling case for *C. gynandra* as a functional food especially for immune-compromised people on homebased care and during the COVID-19 pandemic.

The leaves are the most used edible part of the plant (Van Wyk and Gericke, 2000). Fresh leaves may be dried and stored in a container for consumption later in winter and spring periods. The dried leaves could be stored for about three to four months, or even up to one year (Faber et al., 2010; Steve et al., 2017). In addition, the leaves are being sold by informal street vendors for economic gains (Thovhogi et al., 2021) and this suggests that their value could be on the rise. The cultural beliefs, norms, topography and species availability are believed to influence the extent of its utilisation (Avakoudjo et al., 2019). For instance, Chataika et al. (2020) revealed that the leaves are usually sold in open markets in Namibia and are consumed as food, as well as, being used during various ethnic ceremonies. In Thailand, the leaves are well-preserved as fermented leaf product and consumed afterward (Ekpong, 2009). The leaves and flowers are boiled and consumed as stew, potherb, side dish or tasty relish (Van den Heever and Venter, 2007) (**Table 2**). During preparation, the fresh or dried leaves are usually boiled with small amount of water for about an hour and followed by the addition of oil, onion, tomato, salt and soup powder into the boiling leaves (Faber et al., 2010). The cooked leaves are often consumed with a portion of maize meal- and/or sorghum porridge (Faber et al., 2010). As such, different people prepare it in different forms ranging from boiling to frying.

**Table 2:** Consumption patterns of different parts of *Cleome gynandra* for nutritional food purpose.

Cleome gynandra part	Consumption pattern	Reference
Leaves	Fresh leaves are cooked as vegetables and eaten as stew, potherb, side dish, relish and sometimes as ingredients in other mash foods; dried leaves are ground and incorporated in weaning foods	Mathenge, 1995; Van den Heever and Venter, 2007; Thovhogi et al., 2021
Flowers	Flowers are boiled in stew or as consumed side dish	Van den Heever and Venter, 2007; Faber et al., 2010; DAFF, 2013
Pods, seeds and roots	Uses of pods, seeds and roots as a food source are currently undocumented	

The study carried by Hart and Vorster (2006) showed that people living in the SADC perceived *C. gynandra* leaves to be too bitter for consumption. It was then recommended that the bitter taste can be reduced by either incorporating milk during cooking or changing the cooking water. The leaves can also be mixed with other leafy vegetables such as amaranth, blackjack (*Solanum nigrum* L.) and cowpea (*Vigna* spp.) during cooking preparation to reduce bitterness and increase the bulkiness (Vorster et al., 2002; Van den Heever and Venter, 2007). The origin and extent of bitterness in various accessions of *C. gynandra* necessitate more research enquiries (Moyo and Aremu, 2022).

The concurrent occurrences of COVID-19 pandemic and changing climate have posed an unexpected challenge to the global attempts to tackle food shortage, hunger and malnutrition in all its forms (Development Initiatives, 2021). Considering its nutritional value and health benefits, this leafy vegetable could contribute significantly to the food security and balanced diets of rural households. The utilisation of *C. gynandra* could help in contributing to the achievement of the first three Sustainable Development Goals (1) No Poverty; 2) Zero Hunger and 3) Good Health and Well-being by 2030) (United Nations, 2020). It has the potential to alleviate poverty and nutritional insecurity because it is one of the easily accessible sources of vital nutrients (Kwenin et al., 2011).

## Research gaps, recommendations and considerations

### Prospects on the traditional medicinal use of *C. gynandra*

Recently, various authors provided the rigorous and extensive reviews on updated medicinal and pharmacological information about *C. gynandra* plants as well as their bioactive compounds (Chand et al., 2022; Khuntia et al., 2022; Moyo and Aremu, 2022). Several studies detected the presence of numerous health-promoting phytochemical compounds in *C. gynandra* including alkaloids, flavonoids, glucosinolates, aldehydes, phenols, proanthocyanidins, ketones, saponins, sesquiterpenes, steroid, tannins and terpenoids (Bala et al., 2011; Sowunmi and Afolayan, 2015; Neugart et al., 2017; Moyo et al., 2018). It is reported that these compounds found in *C. gynandra* have high medicinal value and possesses anti-inflammatory, anti-bacterial, antimicrobial, anticancer, antioxidant, antiallergenic, antispasmodic, antihyperglycemic and cytotoxicity properties (Bala et al., 2010; Lawal et al., 2015; Chand et al., 2022). Oboh et al. (2008) also demonstrated that the antioxidant potential extracted from all parts of *C. gynandra* have the capacity to scavenge free radical at different concentrations and thus providing scientific credibility for its therapeutic use as herbal medicine. The consumption of these natural antioxidants has been known to reduce the risks of cancer, cardiovascular diseases, diabetes, inflammation and toxicity (Bala et al., 2010; Chand et al., 2022; Moyo and Aremu, 2022).

The entire plant has been traditionally used in different countries to treat several medical conditions such as anaemia, arthritis, diabetes, cancer, cardiovascular diseases, chest pains, constipation, epilepsy, earaches, malaria, piles, rheumatism, scurvy, stomach-ache, tumour, and relieving eyewash (Faber et al., 2010; Mishra et al., 2011; Moyo and Aremu, 2022). The sap from leaves is used for the management of severe threadworm infections and relieving cerebral pain. Also, the sap from pounded young leaves is squeezed into the ears, nose, and eyes in attempt to control epileptic seizures and relieve earaches (Khuntia et al., 2022). The decoction of leaves and roots is used to relieve fever and headaches as well as alleviate sexual weakness (Bala et al., 2011). Also, the extracts are used in treating snake bites, food poisoning and severe pain caused by scorpion stings (Al-Asmari et al., 2017). In India, it is believed that regular leaf consumption by pregnant women may ease childbirth by reducing dizzy spells and decrease labour duration (Oboh et al., 2008; Mishra et al., 2011). It is also perceived that drinking boiled leaves increase milk production in breast feeding mothers (Dansi et al., 2008).

Indeed, *in-vitro* and *in-vivo* studies carried out on most of the plant extracts support primary traditional uses of the plant species such as *C. gynandra*. Nevertheless, it is important to note that most of the claims reported in this review about traditional therapeutic uses of *C. gynandra* have not been proven through sound scientific investigations on human and animal subjects; and would thus require further research. Therefore, future studies could assess widely the mechanisms of actions of these medicinal uses of the species for the treatment of diseases to validate their effectiveness. Also, clinical trials should be conducted to confirm the safety and efficacy of pharmaceutical products derived from *C. gynandra* in humans.

### Challenges concerning cultivation of *C. gynandra*

Seed germination of *C. gynandra* occurs within five days under normal conditions and seedlings are thinned after three weeks (Onyango et al., 2013). However, poor seed quality and delayed seed germination due to dormancy are the main constraints of propagation of these species (Abukutsa-Onyango, 2007). According to Ekpong (2009), improved seed quality is a prerequisite for successful crop production. Several studies also reported other challenges such as lack of seed availability, poor seedling establishment, low yield and pests and disease susceptibility (Chivenge et al., 2015; Mabhaudhi et al., 2019a). Most orphan crops, including *C. gynandra*, produce poor yields and have inadequate benchmarking when compared to major crops (Mabhaudhi et al., 2019b). These challenges are mainly caused by low investment in breeding and germplasm development as well as poorly established agronomic practices. The fact that *C. gynandra* thrives with little conventional support suggests that it can be useful in adapting to changing climate due to its resilient traits (Mabhaudhi et al., 2019a). Research aiming at improving its seed and yield quality, establishment of appropriate plant densities, planting dates and fertiliser rates as well as pest, disease and weed management strategies are required to ensure optimum sustainable production of this leafy vegetable.

Since they grow naturally in the wild, *C. gynandra* plants had been viewed as weeds (Drimie and Pereira, 2016). As such, there is a need to initiate management practices for this vegetable and promote its cultivation and utilisation. The diversity of agronomic practices on *C. gynandra* has not been explored and its economic importance is relatively unknown. Empirically based information on suitable agronomic practices (i.e., fertiliser and water requirements, planting density, spacing, harvesting age and interval) and management practices need thorough research and development. Moreover, these plants have been observed to have insecticidal, antifeedant and repellent characteristics (see Malonza et al., 1992 and Van Rensburg et al., 2007a for more information). Future research studies should examine this plant protectant characteristic possessed by *C. gynandra* through rigorous empirical trials. This is because findings on these various aspects could expand the knowledge needed to ensure its optimum production and utilisation.

### Potential of breeding *C. gynandra*

Plant species such as *C. gynandra* with extensive geographical occurrence experience diverse environmental conditions and this causes them to evolve in varied morphological and phytochemical structures (Sogbohossou et al., 2018). The necessity for characterisation, conservation and cultivation of *C. gynandra* remains vital in preserving its genetic information integrity and diversity (Munene et al., 2018). Therefore, vigorous promotion of its conservation and intensive use needs to be addressed (Coq-Huelva et al., 2017). Several reports showed that germplasm resources for *C. gynandra* are available in local, regional, and international genebanks (Achigan-Dako et al., 2021). Currently, there are about 295 accessions of these species available at World Vegetable Centre (http://www.seed.wordveg.org/) collected from Southern African, Eastern Africa and Asia (Moyo and Aremu, 2022). However, there is currently a lack of extensive collection and conservation of *C. gynandra* germplasm in countries where they are distributed (Kwarteng et al., 2018). Only few countries have reportedly collected and preserved germplasm of this species. For example, there are 45 accessions of *C. gynandra* comprising 28 landraces, 9 wild, 7 weedy and 1 unknown accessions at the National Gene Bank of Kenya (Kemei et al., 1995). According to Achigan-Dako et al. (2021), 164 accessions of *C. gynandra* germplasm were recently collected from Benin, Burkina Faso, Ghana, Niger and Togo and were maintained at the University of Abomey-Calavi’s Laboratory of Genetics, Biotechnology, and Seed Sciences in Benin. More than a decade ago, it was reported that about 184 accessions of *C. gynandra* were collected in South Africa and Tanzania by National Gene Bank of South Africa and National Plant Genetic Resource Center (Arusha–Tanzania) (Van Rensburg et al., 2007a). According to Wasonga et al. (2015), these large number of collected accessions of *C. gynandra* in the genebanks of countries like South Africa and Kenya lack proper documentation. Moreover, they have not been characterized systematically in terms of their morphology and agronomic variability. The scarcity of information on the collection and conservation strategies in areas where these species is occurring, prompt the need to widely collect and conserve germplasm of *C. gynandra*. This will aid in generating a wider genetic diversity needed for improving the species and adverting genetic erosion.

Within the accessions of *C. gynandra*, in-depth investigations on the morphological characterisation are required to determine their genetic diversity (Kwarteng et al., 2018). This should be done to identify proper materials that could be utilised in cross breeding in order to develop improved varieties with certain useful qualities. The study by Sogbohossou et al. (2019) showed that 76 C*. gynandra* accessions collected from sub-Saharan Africa and Asia have the significant variability in morphological characteristics. For example, the West African accessions has shorter plants with tiny leaves when compared to Asian accessions with short plants and broad leaves, whereas East-Southern African accessions has tall plants. Furthermore, *C. gynandra* collected from East-Southern Africa has low tocopherol content, whereas plants from both Asia and West Africa showed high levels of tocopherol (Sogbohossou et al., 2019). Another study carried out by Kiebre et al. (2015) demonstrated that the phenotypic characteristics of 30 C*. gynandra* accessions from five various ecological locations in Burkina Faso were greatly different from each other. These consists of qualitative characteristics such as stem colour, leaf colour, fruit shape and flower colour, as well as quantitative traits like plant height, stem diameter, leaf size, number of branches, fruit length and leaf biomass.

At the molecular level, *C. gynandra* shows an important genetic variation and this was demonstrated by various markers including random amplified polymorphic DNA, simple sequence repeat, inter simple sequence repeat and single nucleotide polymorphism (Achigan-Dako et al., 2021). The vast genetic variability affords researchers with a wide germplasm for crop breeding programs intended to enhance desired plant characteristics and quality (Shilla et al., 2019). This includes genotype with improved tolerance to biotic and abiotic stresses, optimised leaf yield, flowering time, leaf traits, plant height, extended storability, reduced leaf bitterness and better nutritional quality (i.e., essential minerals, vitamins A and C) (Onyango et al., 2013; Sogbohossou et al., 2019). According to Mathooko and Imungi (1994), bitterness in the leaves of *C. gynandra* is caused by a fraction of phenolic compounds called condensed tannins. Because of their undesirable palatability, these condensed tannins protect the plants against pests (Kutsukutsa et al., 2014). Nevertheless, it is necessary to decrease the levels of these tannins in *C. gynandra* to alleviate bitter taste.

Currently, there are efforts to produce desired crop cultivars that could meet the growing demand of food while at the same time supporting sustainable productivity. As such, improving crops by using new breeding techniques is on the rise globally. These precise and faster genetic crop modifications have been made with ease due to development of biotechnological approaches such as genome editing, genetic engineering and genome mapping (Munaweera et al., 2022). According to Khew et al. (2022), two approaches that are gaining prominence in the fields of molecular biology and plant breeding due to their precision and efficiency include marker-assisted selection (MAS) and genome editing (GE).

Marker-assisted selection involves indirect selection and improvement of desirable traits of interest in a short period through DNA markers (Sakiyama et al., 2014). In plant breeding, it has been broadly used to characterise germplasm, gene pyramiding, genetic purity, diversity analysis, multi-trait introgression and trait stacking of various commercial crops (Kumawat et al., 2020). Because of its high precision, MAS could be used to develop desired *C. gynandra* cultivar with vast agronomic values. Nevertheless, the efficacy of MAS on selection may be hampered by several factors including genetic background, high input cost, insufficient linkage, limited molecular markers, reliability and precision of QTLs as well as their narrow range of polymorphism (Kumawat et al., 2020).

Although not so new, genome sequencing has become affordable in facilitating crop improvement even in resource-poor countries of the world. To date, about 800 plant genomes have been sequenced globally. However, only 20 of these plant species are indigenous to Africa and none have been sequenced within the continent. This is irrespective of the fact that the continent has the second largest plant species after South America (Bafana, 2022). The launching of the African BioGenome Project in 2021 could provide a platform to sequence the genome of numerous endemic plants that are of cultural, economic and scientific significance such as *C. gynandra* (Bafana, 2022). Ongoing projects at the African Orphan Crops Consortium have presently sequenced and published eight genomes; with two more genomes of finger millet (*Elusine coracana*) and *C. gynandra* being assembled (http://africanorphancrops.org/ongoing-projects/). Moreover, the efforts to assemble the genome of *C. gynandra* for providing robust resources to speedily develop better cultivar (Achigan-Dako et al., 2021). These genome data of *C. gynandra* will be beneficial in identifying and characterising genes of agronomic significance and understand their mode of actions as well as allowing breeding strategies for designing intensive, quicker and predictable crop improvement programs. Given the rise of malnutrition cases globally, the germplasm of *C. gynandra* could be valuable to improve human health and combat malnutrition and hunger (Thovhogi et al., 2021). Differences in nutrient concentrations provide opportunities for breeding better cultivars with desired mineral levels (Omondi et al., 2017). Also, it is crucial to consider regulating levels of bitterness in its leaves through breeding to desired consumer preferences (Moyo and Aremu, 2022). Indeed, optimising genetic crop productivity qualities as well as nutritional components are important for producing high-yielding *C. gynandra* cultivars.

Breeding programmes should also aim at exploring the mechanisms involved in the response and adaptation of *C. gynandra* as a C4 plant to salinity stress (Kwarteng et al., 2018). This will help in introducing genetic manipulations targeted at enhancing salinity resistance. Moreover, susceptibility to pests and diseases has also been identified as one of the major production constraints of *C. gynandra*. Chweya and Mnzava (1997) reported that this plant is infested by locusts, nematodes and pentatomids as well as being a host to diseases like powdery mildew. As such, breeding programmes should also aim at improving the *C. gynandra* plants to tolerate or resist such pests and diseases. Through breeding programmes, the development of resistant lines that can be used in the integrated pest and diseases management will reduce the overreliance of pesticides which has negative impact on the environment (Kwarteng et al., 2018).

A more recent breeding technique that is still controversial at the regulatory level but equally a game-changer, is the gene editing (Schmidt et al., 2020). This technique depends on genome modification by inserting, replacing, eliminating or disrupting the DNA sequences using tools such as molecular scissors and artificial nuclease enzymes (Kamburova et al., 2017). Thus, making it more beneficial to both basic and applied sciences. The breakthroughs in genome editing like base editing, CRISPR/Cas9, CRISPR/Cpf1, dCas9 epigenetic modification, prime editing and many other transgene-free genome editing have shown great potential for improving crops (Gowda et al., 2020; Haroon et al., 2022). These improvements include the development of high-yielding cultivars with better stress tolerance and enhanced nutritional values (Khew et al., 2022). Varshney et al. (2021) and Haroon et al. (2022) emphasised CRISPR/Cas9 and Cpf1 are more efficient, precise and accurate and have less off-target effects when compared to other genome editing technologies. Improving the important agronomic traits of *C. gynandra* using these technologies could benefit its potential commercialisation. Indeed, the cost effectiveness, ease, and speed of designing genome editing tools makes it a highly suitable and viable system for crop development (Aglawe et al., 2018).

AUDA-NEPAD is driving the adoption and scaling-up of novel technologies for socio-economic development. In that respect, many efforts have been initiated including “Freedom to Innovate” publication of 2007; the African Biosciences Initiative Networks like Southern Africa Network for Biosciences (SANBio), Science Technology & Innovation Strategy for Africa (STISA 2014-2024) and the formation of the Africa High Level Panel on Emerging Technologies (APET) for priority setting and Africa Biosafety Network of Expertise (ABNE) on supporting member states on regulation (AUDA-NEPAD, 2021). AUDA-NEPAD also exercises leadership in supporting AU member states in international negotiations at the Convention on Biological Diversity Conference and Meeting of the Parties to the Biosafety Protocol (COP-MOP). More recently, AUDA NEPAD established AUDA-NEPAD Centres of excellence on Science, Technology, and Innovation (AUDA-NEPAD CoE-STI) where a genome editing flagship project is being implemented. It is imperative that proper stewardship and scaling-up of the new breeding techniques such as gene editing be used to accelerate crop improvement, especially with orphan crops such as *C. gynandra*.

### Constraints on research development and popularity of *C. gynandra*

Progressive attempts toward reinvigorating indigenous crops in Africa have been made thus far. For instance, Indigenous knowledge system (IKS) funding of South African Department of Science and Innovation (DSI) and National Research Foundation (NRF) made efforts to support research that focuses on IKS, indigenous crops, community development and involvement (NRF, 2018). However, these attempts have been met with several constraints such as lack of value chain of indigenous crops and poor conservation practices (Mudau et al., 2022). Despite the unique qualities of *C. gynandra*, it has received little research attention or extension activities and has been ignored by the international science mainstream (Aworh, 2018; Rugube et al., 2019). Also, traditional leafy vegetables like *C. gynandra* are often considered as ‘famine foods’ eaten by the rural populace during times of major food crop shortage (Omondi et al., 2017).

The study by Thovhogi et al. (2021) showed that participants’ age influenced their dietary preferences, with the old people consuming *C. gynandra* more than the young ones. Indeed, the younger generation is accustomed to the textures and taste of modern vegetables and common fatty foods (Mbhenyane, 2016; Sogbohossou et al., 2018). Van Rensburg et al. (2007a) suggested that promotion activities of African leafy vegetables could change this belief. Nonetheless, access to and knowledge of diverse uses of *C. gynandra* is context-specific due to cultural and societal differences. Therefore, information collected during some studies used in this paper is specific to certain areas and therefore cannot be generalised (Faber et al., 2010). As such, studies should be conducted to completely document local knowledge on consumption patterns and prospects of *C. gynandra* as a food and medicinal source among individuals living in its established populations. This is because the inclusion of local knowledge by communities on the utilisation of certain plant species is a key element to cogitate in decision making about their promotion and development as a prospective cash crop.

Currently, there is no commercial production of *C. gynandra* and it is only available during the rainy season; thus, limiting its consumption frequency (Thovhogi et al., 2021). As such, detailed strategies should be developed to promote its household consumption and potential commercialisation (Maseko et al., 2017). Subsequently, this will increase the chances of introducing it as a future cash crop to farmers as well as helping in the fulfilment of the SDGs of reducing poverty, and ensuring household food and nutrition security by 2030, especially among the rural communities. The SDGs recognise agricultural productivity and rural development as the essential priorities for reducing poverty, addressing food security, job creation and economic growth (United Nations, 2020). The current poor status of this crop may be improved by its development and could positively contribute to sustainable production. The promotion of *C. gynandra*’s production and utilisation could be enhanced by conducting workshops, seminars and community outreach as well as incorporating into government programmes and policies.

### Considerations on postharvest of *C. gynandra*

Rigorous research on different aspects (i.e., nutritional and phytochemical contribution at postharvest) is warranted. Nutritional analysis after cooking and/or processing of *C. gynandra* could be fundamental to marketing as this is vital in offering quality assurance and integrity to consumers. Currently, there is poor development in the value chain of most orphan crops and *C. gynandra* is not an exception (Mudau et al., 2022). Therefore, this leafy vegetable provides many opportunities to expand current food systems and thereby contributing to job creation, food security and poverty alleviation. According to Preston (2014), food systems include all procedures and stages involved in feeding a population. This includes growing, harvesting, post-harvest processing and packing, storage, transporting, marketing, utilisation, and food/food-related items disposal. Postharvest technologies that preserve *C. gynandra* need to be explored to adapt to modern market trends and demands (Mabhaudhi et al., 2019a). Generally, value addition to this crop could provide various prospects for establishing agro-processing industries in marginal communities and subsequently promoting rural economic growth.

## Conclusion

*Cleome gynandra* is an excellent low-cost source of essential nutrients such as vitamins and minerals with greater potential in contributing significantly to health, food diversity and balanced diets of resource-poor communities across the African continent. The plant is an annual, climate-smart crop capable of countering the negative effect of climate change on agricultural production. Although it is widely adapted to tropical and sub-tropical regions, there have been limited efforts towards its improvement for production and consumption. Regardless of its potential benefits, it is currently underutilised and neglected worldwide. The development of this cultigen into cultivated crop is still at infant stage due to lack of knowledge about its availability, affordability, nutritional value and health promoting properties. Also, paucity of endorsement on this vegetable hampers its production on a larger scale. As such, the need to develop management practices for this leafy vegetable and encourage its cultivation, commercialisation and utilisation should be prioritised.

In this review, we highlighted the prospects of producing desired cultivars of *C. gynandra* with improved tolerance to biotic and abiotic stresses, optimised leaf yield, better nutritional quality and reduced bitterness. Innovation on diversity of processing techniques and value-added products are key aspects to consider for the development of value chain of this leafy vegetable. Sun-drying and cooking are the most post-harvest processes used in Africa. Further research should be conducted to identify better and faster drying methods that maintain the nutrient quality of *C. gynandra* while at the same time expanding its value- added products, using modern food processing technologies such as canning, extrusion, flaking, malting and rolling. Diversifying new products could promote its acceptability, consumption and demand by rural and urban communities. This will ensure that its quality products are well positioned for trading in the formal and international markets.

## Author contributions

CM and AM: conceptualisation. CM: writing − the initial draft; writing – revisions. AM and EC: writing − review and editing. AM: funding acquisition and project leadership. All authors contributed to the article and approved the submitted version.

## Funding

This research work was supported by Govan Mbeki Research and Development Centre of the University of Fort Hare (South Africa).

## Conflict of interest

The authors declare that the research was conducted in the absence of any commercial or financial relationships that could be construed as a potential conflict of interest.

## Publisher’s note

All claims expressed in this article are solely those of the authors and do not necessarily represent those of their affiliated organizations, or those of the publisher, the editors and the reviewers. Any product that may be evaluated in this article, or claim that may be made by its manufacturer, is not guaranteed or endorsed by the publisher.
